# Health care provider reporting practices related to self-managed abortion

**DOI:** 10.1186/s12905-023-02266-7

**Published:** 2023-03-27

**Authors:** Sarah C. M. Roberts, Claudia Zaugg, Daniel Grossman

**Affiliations:** Advancing New Standards in Reproductive Health, Department of Obstetrics, Gynecology and Reproductive Sciences, University of California, San Francisco, 1330 Broadway, Suite 1100, Oakland, CA 94612 USA

**Keywords:** Abortion, Reproductive Health, Healthcare Provider Perspectives, United States

## Abstract

**Background:**

Health care providers reporting patients to government authorities is a main way people attempting self-managed abortion (SMA) become exposed to legal risks. Little is known about health care provider decision-making regarding SMA reporting.

**Methods:**

We conducted semi-structured interviews with 37 clinicians who provided care in hospital-based obstetrics or emergency departments (13 obstetricians/gynecologists, two advance practice registered nurses providing obstetrics care, 12 emergency medicine physicians, and 10 family medicine physicians) throughout the United States. The interview guide asked participants to describe one or more cases of caring for a patient who may have attempted SMA and about related reporting decisions. We coded responses to answer two questions: What comes to mind for health care providers when asked to think about experiences caring for a patient who may have attempted SMA? Based on health care provider experiences, how might people who providers suspect may have attempted SMA end up reported?

**Results:**

About half of participants had cared for someone who may have attempted SMA for that pregnancy. Only two mentioned SMA with misoprostol. Most participants described cases where they were unsure whether the patient had attempted to end their pregnancy on purpose. In most instances, participants mentioned that that the possibility of reporting never occurred to them nor came up. In some cases, participants described a reporting “adjacent” practice – e.g. beginning processes that could lead to substance use, domestic violence, or self-injury/suicide-related reports – or considered reporting related to a perceived need to report abortion complications. In two cases, hospital staff reported to the police and/or Child Protective Services related to the SMA attempt. These involved passing of a fetus after 20 weeks outside the hospital and a domestic violence incident.

**Conclusion:**

Reporting patients who may have attempted SMA may occur via provider perception of a need to report abortion complications and fetal demises, particularly at later gestations, and other reporting requirements (e.g. substance use, domestic violence, child maltreatment, suicide/self-harm).

## Background

In the aftermath of the 2022 *Dobbs vs. Jackson Women’s Health* U.S. Supreme Court decision that allows individual U.S. states to make abortion illegal again, more people in the U.S. will likely attempt to self-manage their own abortions (SMA). Unlike before 1973, the previous era in which abortion was illegal in many U.S. states, there are now safe and effective SMA methods. These safe and effective SMA methods include self-sourced abortion medications (misoprostol used alone or together with mifepristone) [1–3] that people obtain online or from other countries where these medications are available. Not everyone uses these methods when they seek to end their pregnancies on their own, though, [4, 5] and some methods may be less safe and effective than SMA with self-sourced abortion medications. [3].

Regardless of which SMA method people use, a primary risk with SMA is legal risk, as laws in some U.S. states criminalize SMA. [6] Even without explicit laws on the books criminalizing SMA, people in the U.S. have been prosecuted and imprisoned for self-managing their abortions. [6, 7] Importantly, a study of arrests and forced interventions on pregnant women in the U.S. from 1973 to 2005 found more than 400 cases that resulted in arrests, detentions, and forced interventions on pregnant women, some of which were related to people ending their pregnancy on their own. [8] A more recent study identified 61 cases between 2000 and 2020 of people criminally investigated or arrested for allegedly ending their own pregnancy or helping someone else do so. [9] People of color and people further along in gestation comprised larger shares of those investigated or arrested and people living in poverty comprised a larger share of those prosecuted. [9] Across both studies, the biggest source of reports to law enforcement was health care providers. [8, 9]

While there have been a few studies about health care provider decision-making about reporting pregnant and birthing people to government authorities [primarily to Child Protective Services (CPS)] related to substance use during their pregnancies, [10–13] only one study has explored health care provider reporting to government authorities (e.g. CPS, the police, or the health department) in the context of SMA. [14] This study examined perspectives and experiences of hospital-based clinicians’ based in the Texas/Mexico border area caring for patients presenting for care with early pregnancy complications, possibly related to SMA. Of health care providers interviewed, more than half saw no need to report suspected SMA to police or other government authorities. Sometimes, though, social workers became involved and this sometimes led to reports to CPS or police. These reports occurred related to protecting minors from coercion or abuse and in reaction to potential harm to a viable fetus and were more common at later gestations. This study, while an important contribution to our understanding of how health care providers come to report people self-managing abortions to government authorities, focused on one small geographic area of the country.

Health professional association statements oppose criminalization of SMA. [15–18] Recent guidance from the U.S. Health and Human Services Office of Civil Rights explains that reporting patients who have attempted a self-managed abortion to law enforcement, in most instances, violates federal laws protecting patient privacy. [19] While some states’ requirements to report abortion complications to the health department include reporting whether the complication resulted from a SMA, based on legal research to date, no state laws require reporting of SMA to law enforcement (personal communication with If/When/How., on January 18, 2023). However, only very limited research [14] has explored how health care providers may come to report people who seek to end their pregnancy on their own to government authorities. In this study, we seek to expand our understanding of how health care providers come to report people who seek to end their pregnancies on their own. Using data from in-depth interviews with health care providers who provide care in hospital labor and delivery and postpartum units and in emergency departments (ED) throughout the U.S., we explore answers to two questions relevant to understanding how health care providers come to report people who seek to end their pregnancies on their own:

- What comes to mind for health care providers when asked to think about experiences caring for a patient who may have attempted to self-manage an abortion?
- Based on health care provider experiences, how might people who providers suspect may have attempted to self-manage an abortion end up being reported to government authorities?

Exploring these questions can inform future interventions with health care providers to reduce legal risks for pregnant people.

## Methods

Between March – July 2021, we conducted in-depth interviews with clinicians who provide care in hospital-based obstetrics or emergency departments to understand how they make decisions about reporting pregnant and birthing people to government authorities related to their behaviors during their pregnancy or their pregnancy outcomes. As part of that study, we focused on their decision-making both related to pregnant people’s alcohol and/or drug use and related to SMA. We have previously published findings related to their alcohol/drug-related decision-making. [13] In this manuscript, we focus on the SMA-related decision-making.

Study methods have been described in detail elsewhere. [13] The protocol was reviewed and approved by the University of California, San Francisco Institutional Review Board. Briefly, people were eligible to participate if they were 18 or older, currently worked in a hospital-based labor & delivery or postpartum unit or emergency department, and had cared for at least one pregnant person who had used alcohol and/or drugs or at least one pregnant person who they thought may have self-managed an abortion in the past year. We purposively sampled physicians from a range of U.S. geographic regions, including both urban and rural areas, as well as a range of specialties (obstetrics/gynecology, family medicine, and emergency medicine). We initiated recruitment via emails that the study team sent to colleagues, who then forwarded the emails to other individuals, listservs, and physician social media groups. To ensure a sample that included people from different specialties and geographic regions, we tracked participation by region, geography, and specialty. After completing about one-third of interviews, we modified our recruitment strategy to send targeted emails to recruit a more balanced sample. Fifty-seven people completed the eligibility screener; we attempted to schedule interviews with 46 of those who completed the screener and completed interviews with 37. Participants were from eight out of nine U.S. census regions, slightly more from the East North Central region and no participants from the East South Central region, and nine were from rural areas. Participants included 13 obstetricians/gynecologists, two advance practice registered nurses providing obstetrics care, 12 emergency medicine physicians, and 10 family medicine physicians.

We used an interview guide informed by the Theoretical Domains Framework, an implementation science framework to inform provider behavior change. [20] This framework synthesizes psychological constructs from a wide range of health care provider behavior change theories to inform health professional behavior-change interventions. [20] The interview guide included in-depth discussions of one or more cases of either pregnant people’s use of alcohol and/or drugs or of SMA and how decisions about reporting were made for those cases as well as views on and reflections on reporting to government authorities in general. It included questions relevant to the Theoretical Domains Framework, e.g. beliefs about consequences, social/professional role and identity, and emotion regulation. [21] In relation to SMA, we offered a definition of SMA at the beginning of the interview. The definition was “By self-managed abortion, I mean an abortion without clinical supervision, for example with pills people obtain online, herbs, hitting oneself in the stomach. I’m not talking about medication abortion where people get the medications through care from a clinical professional.” We asked participants which experience – caring for a pregnant person using alcohol and/or drugs or a person who may have attempted SMA – was more common for them. We started the interview with the more common experience, which, in all cases, was a pregnant person who used alcohol and/or drugs. After going through the alcohol and/or drug-focused cases and experiences, we then asked participants to think about a patient they had cared for who may have attempted SMA and to describe the case. We then asked whether the possibility of reporting ever came up in the SMA case and, if so, for the participant to tell us what happened.

The first author conducted all interviews, which lasted a median of 44 min, and took careful debrief notes after each interview. Participants were remunerated with a $50 gift certificate for their time. All interviews were audio-recorded and then professionally transcribed.

For the analyses, the first and second authors read through all transcripts. The authors tagged the portions of the transcripts that addressed SMA and, as part of our analyses applying the Theoretical Domains Framework to the entirety of the transcripts, deductively coded the descriptions of the SMA cases with this framework. We had initially intended to follow the guidance for using the Theoretical Domains Framework for analyses [21] as we did for our analyses of physician decision-making regarding reporting pregnant people who use alcohol and/or drugs. [13] However, caring for patients who had attempted SMA was rare enough in the data that deductively applying the framework was not feasible or appropriate. Instead, using data from the participants who could recall a case that they believed may have been an SMA attempt, we explored answers to two questions:

1. What comes to mind for health care providers when asked to think about experiences caring for patient who may have attempted to self-manage an abortion?
2. Based on health care provider experiences, how might people who providers suspect may have attempted to self-manage an abortion end up being reported to government authorities?

To explore the answers to these questions, we categorized the type of SMA method participants mentioned and coded themes that emerged related to reporting. The authors reviewed preliminary findings together and got feedback from a Community Advisory Board, comprised of people with lived experience relevant to a series of studies related to pregnant people’s substance use and/or to SMA.

## Results

About half (n = 18) of participants had cared for someone who they believed might have attempted to self-manage an abortion. Only two mentioned SMA attempts with abortion medications, with one specifying that the medication was misoprostol. Most of the cases were cases where the participant was not sure whether it was an SMA attempt or not. Participants described two cases where they were directly involved in the care of someone where the hospital reported the person to the police and/or to CPS related to the SMA attempt. They also described many reporting “adjacent” practices – i.e. practices that initiated other hospital processes related to the particular SMA method and could lead to reports related to the particular method– and reporting pathways that were considered or initiated.

### What came to mind

When asked about patients who may have attempted SMA, participants described both ambiguous situations where it was not clear whether the person had attempted to end their pregnancy on their own and cases where it was clear that a person had tried to end a pregnancy on their own. They described both specific patients as well as what we term “composite cases” where providers described a typical or common scenario based on more than one patient, without focusing on particular details of any individual patient.

Ambiguous situations involved the following: injury/trauma, infection, suicide attempts, bleeding/miscarriages, homeopathic substances, uterine rupture, and alcohol/drugs [See Table 1].

**Table 1:** Ambiguous Cases: Patients who may have attempted to end their pregnancy on their own

Presentation	Specific cases	Composite cases
Injury/ trauma	Woman, maybe “on drugs,” came into ED as an “auto verse[s] pedestrian” but it was not clear from Emergency Medical Services whether/how she got hit. “I didn’t know…if she had tried to hit herself.”	“trauma patients I suspected were either abused or purposefully caused the trauma to try to end the pregnancy, but…would not confirm”
	Woman “came into triage for…falling, and there was some suspicion that maybe it was self-inflicted.”	
	Patient’s “husband beat her in the stomach. To try to induce an abortion.”	
Infection	Woman “delivered…pre-viable baby and was very sick and in the [Intensive Care Unit].” She “wouldn’t give any history of any kind or allow any records to be obtained from anyone anywhere.” She “delivered and started getting better.” “The whole consultation of things made people concerned that something might have happened potentially related to the pregnancy and trying to end it that had made her so sick.”	“All of our septic [abortions] that come in…we don’t really know what happened.”
Suicide attempt	Suicide attempt among “college freshman from a super religious family who had started college, been pregnant over the summer, and been in denial about it;” had maybe taken some pills earlier in the pregnancy to try and “resolve it, even though she had not really admitted to herself that she was pregnant.”	Some patients early in pregnancy and “have come in suicidal and maybe taken a handful of Tylenol or something”
Bleeding/ miscarriage	“The patient came in through the ED via [emergency medical services] with vaginal bleeding several days after reporting passing an 18 or 19-week fetus at home that she reported had like self-disposed of the remains.” Questions about pregnancy duration, as [last menstrual period] & placental size suggested it was later in gestation. “There was concern both about, you know, what might’ve gone on after what was presumed to be like a fetal demise. And/or if the patient may have induced an abortion.”	Caring for patients who reported doing something to “make my period come down”
	Patient came into ED hemorrhaging profusely, reporting a 1st -trimester miscarriage. “Maybe that truly was what was going on, but I had never seen anything like that. It was - the story was, ‘Oh, I was fine, and just like in an hour I was in a bathtub and all of the sudden I started hemorrhaging.”	
	Person came into ED stating miscarriage at 16 weeks; her chart (from previous visit) said she had been considering abortion. Came into ED and passed “intact fetus” pretty quickly.	
	Homeless woman, who was likely intoxicated, came into ED holding her fetus.	
Homeopathic		“Some of them, I think, tried homeopathic stuff.”
Uterine rupture	“She…had a uterine rupture and ended up in the [operating room]…she didn’t tell either me or the [obstetrician] that she had had an abortion…her pregnancy test was positive, she was unstable, and she was bleeding into her abdomen…She definitely did not tell us anything about the abortion. The [obstetrician] sonly learned later because she saw like the [unintelligible] sites.” “She was acutely ill, too. Like if she hadn’t gone to the OR she would’ve died.”	
Alcohol/ drugs		“poor health behaviors that they were hoping would cause the baby to demise”
		Someone early in pregnancy in ED who had “tried to overdose on something in order to have an abortion”

Whether these were cases in which a patient intentionally sought to end their pregnancy on their own may be less relevant for reporting decisions than what providers think about the ambiguous situations. For some participants, there were aspects of what happened that made them wonder whether the person had done something on purpose to try to end the pregnancy. Some participants used language that reflected more of an investigative or perhaps even law enforcement framework to describe what made them think that it was possibly an SMA attempt:

*“Like, the story seemed sketchy, you know, like ‘Did you do that on purpose?’” – Emergency Medicine*

*“There was some suspicion that maybe it was self-inflicted. But, it’s…hard to say that’s what really happened.” – Obstetrics/Gynecology*

*“There was concern that the like placental size did not match up and that the patient’s story was a little bit variable depending on with whom the patient was speaking.” – Family Medicine.*

In a small minority of cases, providers probed to try to assess what might have happened. For example, one participant mentioned directly asking a patient who had presented with a septic abortion “how all this came about.” In more cases, though, providers described being comfortable with the uncertainty and ambiguity and acknowledged that whether there was an SMA attempt or not was not relevant to the clinical care.

*“In those cases, we don’t ask where it was done. Or if we do, they don’t remember. So, you know, those are always curious situations.” – Obstetrics/Gynecology*

*“So I think that’s where I always sort of allow a little bit of maybe.” – Emergency Medicine*

*“There was something between us that I could just tell that maybe this was not something that she was totally unhappy about it.” – Family Medicine.*

The types of cases where participants were confident that the person had tried to end their pregnancy on their own generally fell into similar, but fewer, categories in terms of SMA method as the more ambiguous cases. The most common method described was people using alcohol/drugs; participants also mentioned injury/trauma, medication abortion, and homeopathic substances. [See Table 2]

**Table 2:** Clear Cases: Patients who had tried to end their pregnancy on their own

Presentation	Specific cases	Composite cases
Injury/ trauma	“a patient asked her boyfriend to punch her in the stomach”	
Medication abortion	Person presented in ED “septic from an incomplete abortion.” Disclosed had taken abortion medication her cousin had mailed to her from Caribbean	Multiple patients had obtained misoprostol online or “gone to Mexico to retrieve it”
Homeopathic	Came into abortion clinic because homeopathic substances didn’t work	
Alcohol/ drugs	Tried in 1st & 2nd trimester to “induce a miscarriage with alcohol and drugs, and then it didn’t work.” Decided to continue pregnancy. Disclosed when presented in [obstetrics] triage because concerned about effects on baby.	
	Patient admitted for substance use disorder treatment in 2nd trimester; as part of [obstetrics] history, mentioned that had tried to “stop [a previous] pregnancy by…taking too much alcohol”	
	Patient “on opioids” entering prenatal care who “did not want to be pregnant” had tried to take a [benzo] to try to “stop the pregnancy”	
	Patient with an opioid use disorder who also used crack/cocaine who tried to induce early labor by using additional cocaine & “actually succeeded”	

### Reporting to government authorities

#### Reporting did not come up

In almost all cases of possible SMA attempts, the possibility of reporting to government authorities did not come up.

*“It did not even occur in my mind…I wouldn’t know what government agency to report it to, but it would never occur to me to report them for that.” – Obstetrics/Gynecology*

*“I’ve never had to deal with anything like legal with that. I’ve never reported that. I’ve never been asked by anyone legal about that…I’ve never even really thought about it.” – Emergency Medicine*

Other participants went a step further and emphasized that, while it had not come up, even if it did, they would not make a report. Their intentions to not report were strong.

*“I don’t think there was anything to report. It’s not [a] state where that kind of thing would be reportable, I don’t think. Even if it was, let’s say I was practicing in Alabama. I would never report something like that personally…I would never report that.” – Family Medicine*

*“I don’t think I would ever report someone if they successfully, so to speak, caused a miscarriage. I don’t think I would report them for that because I think it’s really hard to…guess someone’s motivation for using substances. You know, it could just be that they were super stressed out and that’s how they cope.” – Obstetrics/Gynecology*

#### Reporting adjacent practices

In other cases, people at the hospital considered making a report or engaged in what can be described as a reporting “adjacent” practice. These were not for anything that participants described as abortion- or SMA-related reporting requirements. Instead, these practices were connected to what participants described as reporting requirements related to the possible SMA method: substance use, self-injury/suicide, and domestic violence. For example, in relation to substance use-related reporting requirements in the hospital, a participant described having to fill out a form after a patient entering prenatal care disclosed having used alcohol/drugs to induce a miscarriage. The participant, an obstetrician/gynecologist, mentioned a hospital requirement to fill out this form “*if there’s any report or evidence of [substance] exposure in pregnancy”* and went on to describe that this form *“triggers you to kind of explain what will happen after birth too*.” Another explained,

*“it doesn’t usually work that way, that using lots of drugs and alcohol will actually cause an abortion, so they usually end up continuing the pregnancy because they can’t afford a real abortion. And so, they end up getting reported [to CPS] at the end of their pregnancy because they used drugs during the pregnancy.” – Obstetrics/Gynecology*

One participant described composite cases where someone early in pregnancy comes in after overdosing and where it is possible it was an attempt to end the pregnancy. She then reflected on what would happen if this was determined to be a self-injury/suicide attempt,

*“Not unless there’s a clear indication of a suicide [attempt]. But even then, I mean if it was clear that that’s what was happening, then as an ED doc, I know that I’m not the last one to see that patient. So, if there was an attempt of self-injury, then I know that psychiatry would be involved, or other services. So, I don’t think I would be making that particular decision. And…I would assume that other people would [make the report].” – Emergency Medicine*

This reflection indicates that noting a possible attempt to end a pregnancy as a self-injury/suicide attempt could end up with the person reported to government authorities for that reason.

Another participant described something similar related to domestic violence reporting requirements being invoked. After describing multiple examples of caring for patients with *“either abuse or purposefully cause the trauma to try to end the pregnancy,”* the Family Medicine physician mentioned that *“if someone ever confirmed domestic violence, obviously, that would get a report.*”

These were instances where a report might be made related to the method used for the SMA-attempt. The participants did not mention the possibility of reporting because these may have been attempts to end a pregnancy, but rather because a patient engaged in or experienced a different reportable behavior (e.g. substance use in pregnancy, self-harm/suicide, domestic violence).

A few participants also mentioned possible abortion complications reporting requirements and requirements related to disposal of fetal remains. Specifically, one participant, an Emergency Medicine physician, described, “*In [midwestern state], if you came to me and you were telling me that you were – had self-managed an abortion, we had reporting requirements for abortion complications, and I would imagine those would be triggered with whether they were self-managed or under a clinical supervision.”* This same participant mentioned the possibility of reporting related to requirements related to burial of fetal remains.

#### Actual reports to government authorities

Abortion complication reporting requirements and reporting requirements related to disposal of fetal remains are also illustrated by a case that one participant described. This was not a case the participant had been directly involved with, but rather one that he had heard about from others. In this case, which occurred in a midwestern state, the participant, an Obstetrician/Gynecologist, described a patient undergoing a facility-based second-trimester abortion. *“There was an incident where the police were called for a woman who ended up…trying to undergo a second trimester termination. [She]…was going to go back for a procedure the next day, but ended up slipping into spontaneous labor and expelled the fetus in a…motel.”* According to the participant’s recollection, the patient called 911. *“And then, I think, when 911 got to the scene, they were – because they saw – right – like the fetus. And the just – I think they just called the police out of like – I can’t quite understand [if this was] an abundance of caution.”*

Participants mentioned only two cases, both of which occurred in midwestern states, in which people in the hospital made reports to government authorities related to the SMA attempt. In the first case, Emergency Medical Services brought a patient to the emergency department with vaginal bleeding that continued several days after the patient reported passing a mid-second trimester fetus at home. The patient was then transferred to obstetrics, where the participant was working. Based on other information the patient shared about her last menstrual period date with other people involved in her care, as well as characteristics of the placenta, some people involved in her care thought that perhaps she was closer to the end of the second trimester and thus there might be legal questions related to disposal of fetal remains for pregnancies after 20 weeks gestation. They also considered reporting related to the patient’s older child.

“*The nursing staff was significantly concerned about legal reporting requirements from, and kind of the hospital liability for not reporting someone who had disposed, I think yeah, illegally disposing of [fetal] remains if the patient had been above 20 weeks here. So, there was a question as to whether to call the police directly or to call the [child welfare] system for the care of an older patient, which was clarified to not be a concern that there was a like living child from this pregnancy, but rather from the patient’s older child.” – Family Medicine*

Physicians made the child welfare report for the older child and CPS took the report as a “notification.” Since there was no open case for the older child, nothing further happened. People at the hospital also made a report to police. This came after people were “*uncomfortable with the initial plans that we made*” and “*ended up being a lot more nurse and hospital administrator driven as it was run up the chain.*” The report to police was “*driven by the other factors in the hospital*.” There was not a hospital policy “*that would’ve applied to this case in particular. It was more, you know, individual people, and managers, and liability officers, and the like, and the administration*.”

The second case that was reported involved a person who came in for care in the middle of the second trimester, “*whose husband beat her in the stomach. To try to induce an abortion. But it wasn’t, you know, it wasn’t her that had tried to induce abortion. She was abused throughout her marriage*.” This patient was experiencing ongoing violence from her husband, and the husband was making threats of harming their other child. The participant, a family medicine physician,

*ended up getting authorities involved right away…We were able to get the woman into a shelter and safe space, along with her daughter, away from the father. And we were able to file a police report against the father*…*The father had a warrant that was already out for his arrest. And so, we were able to locate him and get him arrested*.

While in the previous example, the participant expressed concern with what happened in terms of the reporting, in this one, the participant seemed to feel satisfied with the report and the resulting law enforcement action.

## Discussion

Self-managed abortion with self-sourced abortion medications generally was not what came to mind when providers were asked to think about a patient they have cared for who may have attempted to self-manage an abortion. This finding is consistent with recent research that suggests that SMA with self-sourced abortion medications is not necessarily the most common way that people seek to end pregnancies on their own in the U.S. [5] This finding is also consistent with the research that indicates that SMA with self-sourced abortion medications is safe and effective, [22] i.e. few people using these medications to end a pregnancy on their own are likely to present for hospital-based care. This finding, combined with the idea that there are reporting requirements related to some of the SMA methods that came to mind for providers in our study, also suggests that a primary focus on SMA with abortion-medications and on SMA-specific reporting in provider guidance and trainings may miss the other pathways through which providers who care for people who may have attempted an SMA may bring them to the attention of government authorities. These pathways include other reporting requirements (e.g. for substance use, domestic violence, child maltreatment, suicide/self-harm).

Our study identified a small number of cases where health care providers actually reported someone attempting to end a pregnancy on their own to government authorities. Our study also identified multiple pathways through which providers might end up reporting someone who sought to end a pregnancy on their own to government authorities. A few of these cases and pathways were related to abortion-specific reporting, such as provider perception of a need to report some abortion complications and some fetal demises, particularly at later gestations. The abortion reporting requirements that participants mentioned were distinct from the more general reporting requirements that apply to the facility and clinician providing the abortion, such as tracking the number, timing, and type of abortions. [23] However, abortion-specific reporting pathways were not the only possible pathways. Similar to findings from the other study on this topic, [14] reporting of possible SMA to government authorities may end up occurring via other reporting systems. In this study, we identified hospital-based reporting practices related to substance use during pregnancy, domestic violence, possible maltreatment of existing children, and suicide/self-harm as possible pathways through which reporting of SMA to government authorities might occur. A key implication of this study is that only focusing on reporting requirements related to SMA with abortion medications early in pregnancy and not on the broader set of reporting requirements and practices may miss many of the ways that health care providers may bring people who may have sought to end a pregnancy on their own to the attention of government authorities.

Multiple health professional association statements oppose criminalization of self-managed abortion. [15–18] A few health professional statements guide health professionals to not report [3, 24] and the U.S. Department of Health and Human Services Office of Civil Rights has issued guidance indicating that such reporting, when not explicitly required by law, is a HIPAA violation. [19] Professional guidance also instructs clinicians to only ask about and document the possibility that someone did something to attempt SMA in the medical record if it is directly relevant to the clinical care. [3, 25] Yet, such professional guidance does not necessarily translate into practice. While the American College of Obstetricians and Gynecologists has opposed reporting of pregnant and birthing people’s drug use to government authorities for more than a decade, [26] health care professionals, including obstetricians and gynecologists, report tens of thousands of babies to CPS each year related to pregnant and birthing people’s drug use. [27, 28] Even for providers who want to follow this professional association guidance, other factors – such as pressure from others in the hospital, hospital protocols, and a professional obligation to follow existing laws – make it difficult. [13] Guidance, training, and support that reflects experiences of providers navigating these factors and is relevant to the ways their patients are attempting to end their pregnancies on their own seems important. Such guidance and training might include content on the importance of sitting with the ambiguity, or, as one of the participants said, to “always…allow a little bit of maybe,” and to not take on an investigative role of trying to gather more information, as some participants seem to be doing in some cases.

Research on pregnancy-related reporting to government authorities in general and existing research on criminalization of people for SMA consistently shows that health care professionals are more likely to report Black and Indigenous people to government authorities, and government authorities are more likely to prosecute people of color. [9] It is worth noting, though, that participants mostly did not mention racism in their discussions of SMA or SMA-related reporting. This was in stark contrast to the ways providers in this sample mentioned racism related to each step of the reporting decision-making process related to pregnant and birthing people’s drug use. [13] It seems plausible that racism did not come up as part of the discussions around SMA because of the few experiences participants had to reflect on. At the same time, even when these same participants were able to name the range of ways that racism shows up related to reporting of pregnant and birthing people’s drug use to government authorities, they did not have clear solutions for addressing it. [13] It is also worth noting some similarities between the early media coverage of SMA that highlighted it as an exotic behavior among Latin American immigrants [29] and the way the media created the image of “crack mothers” as “women who continued to use crack during their pregnancies… poor women of color, inner-city residents who…care more about crack than about their pregnancies or children.” [30] The racist images of “crack mothers” and “crack babies” have played a central role in policies related to pregnant people’s substance use. [31, 32] Future research should thus explore providers’ understanding of how racism may show up in hospital-based health professional responses to people seeking to end pregnancies on their own.

There are a few limitations worth noting. First, participants had a small number of experiences caring for people who they viewed as having attempted to self-manage an abortion. This is not surprising, given how rare the need for health care is after SMA with medications or herbs is likely to be, and that SMA that effectively ends a pregnancy early in gestation may be clinically indistinguishable from spontaneous pregnancy loss. Second, there were fewer participants from the South, so this may not reflect the full range of physician experiences, particularly in places where prosecutions have been more common. [8, 9] Third, interviews focused primarily on substance-use related reporting. Thus, the interviews may have primed participants to focus more on possible attempts to end pregnancies via alcohol/drugs than they would have otherwise.

## Conclusion

Reporting patients who may have attempted SMA may occur via provider perception of a need to report abortion complications and fetal demise, particularly at later gestations, and other reporting requirements (e.g. substance use, domestic violence, child maltreatment, suicide/self-harm). Focusing primarily on abortion-specific reporting requirements may miss many of the ways in which health care provider reporting behavior may bring people who may have sought to end a pregnancy on their own to the attention of government authorities.

## Data Availability

The datasets generated and/or analyzed during the current study are not publicly available due to privacy and confidentiality concerns. Deidentified portions of the datasets are available from the corresponding author on reasonable request.

## Abbreviations

SMA: Self-managed abortion
CPS: Child Protective Services
ED: Emergency Department
